# Technical Aspects on the Use of Ultrasonic Bone Shaver in Spine Surgery: Experience in 307 Patients

**DOI:** 10.1155/2016/8428530

**Published:** 2016-04-18

**Authors:** Derya Burcu Hazer, Barış Yaşar, Hans-Eric Rosberg, Aytaç Akbaş

**Affiliations:** ^1^Department of Neurosurgery, Faculty of Medicine, Muğla Sıtkı Koçman University, 48000 Muğla, Turkey; ^2^Çankırı State Hospital, Department of Neurosurgery, 18000 Çankırı, Turkey; ^3^Lund University, Skane University Hospital, Department of Translational Medicine, 205 02 Malmö, Sweden

## Abstract

*Aim*. We discuss technical points, the safety, and efficacy of ultrasonic bone shaver in various spinal surgeries within our own series.* Methods*. Between June 2010 and January 2014, 307 patients with various spinal diseases were operated on with the use of an ultrasonic bone curette with microhook shaver (UBShaver). Patients' data were recorded and analyzed retrospectively. The technique for the use of the device is described for each spine surgery procedure.* Results*. Among the 307 patients, 33 (10.7%) cases had cervical disorder, 17 (5.5%) thoracic disorder, 3 (0.9%) foramen magnum disorder, and 254 (82.7%) lumbar disorders. Various surgical techniques were performed either assisted or alone by UBShaver. The duration of the operations and the need for blood replacement were relatively low. The one-year follow-up with Neck Disability Index (NDI) and Oswestry Disability Index (ODI) scores were improved. We had 5 cases of dural tears (1.6%) in patients with lumbar spinal disease. No neurological deficit was found in any patients.* Conclusion*. We recommend this device as an assistant tool in various spine surgeries and as a primary tool in foraminotomies. It is a safe device in spine surgery with very low complication rate.

## 1. Introduction

Spinal surgery has improved with the introduction of operating microscopes and high-speed drills. Occasionally, a surgical approach requires microscopic bone dissection. However, the spinning and increased heat production when drilling with diamond burrs under the microscope may cause damage to the soft tissues such as the dura, nerve roots, the cord, and vessels.

Ultrasonic bone removers have been used for skull base surgery for several years [1] and they have been introduced to spinal surgery recently [2–6]. The most frequently used bone remover is “scalpel-type” ultrasonic bone curette. It has a thin (0.7 mm) and somehow wider tip, resembling a tip of a knife, which is available only for cutting bone. It creates a narrow incision in the vertebral arch for laminectomy and splitting laminoplasty [7–10]. However, due to its tip the “scalpel-type” ultrasound device is not used to remove osseous spurs or ossified lesions when decompressing the nerve roots. We report our experience and discuss the technique with the ultrasonic bone remover used with a microhook tip in cervical, thoracic, and lumbar spine surgeries.

## 2. Materials and Method

### 2.1. Subjects

From June 2010 to January 2014, 307 subjects with various spinal disorders were operated on using the ultrasonic bone shaver (UBShaver). Data from each subject were collected retrospectively from the hospital files. The patient's demographics, disease type, type of surgery, complications, preoperative Oswestry Disability Index (ODI) and Neck Disability Index (NDI), and one-year follow-up ODI and NDI scores were recorded.

The number of patients, level of disorder, type of pathology, and surgical technique are presented in Table 1.

All patients have given their informed consent for participation in this study. This study has been approved by the local ethical committee of Muğla Sıtkı Koçman University Ethical Committee-Clinical Research Section (BAP-02342014).

### 2.2. Instrument

**Figure 1:**
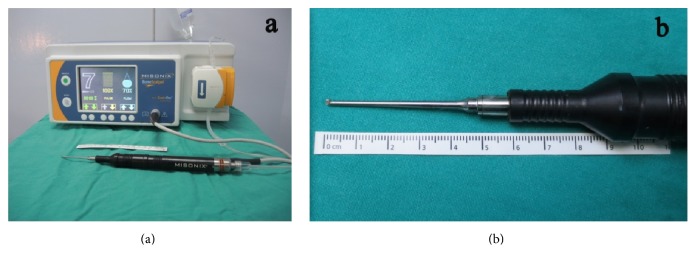
(a) Misonix device with irrigation equipment and a straight hand piece and (b) tip of the Misonix ultrasonic bone shaver are illustrated.

We have used Misonix (MXB-S1, Farmingdale, New York, USA) as an ultrasonic bone curette which is designed for precise removal of rigid bone while remaining atraumatic to soft tissues underneath. The hand piece has an interchangeable tip with an irrigation jet nozzle. The tip of the instrument oscillates in linear fashion back to front at the frequency of ultrasound. It uses a piezoelectric transducer to convert electrical signal into a mechanical vibration. Micromovements are produced at the frequency of 22.5 kHz which only cuts mineralized tissue. We have used the microhook shaver tip (MXB-S1) which is 1.8 mm in width, with a short extension and a silicon cover (Figures 1(a) and 1(b)). The surgical device used at our unit comprised a power supply with a footplate and a straight hand piece.

### 2.3. Surgical Techniques

#### 2.3.1. Lumbar Posterior Applications

The most frequent application of the UBShaver is for posterior lumbar surgery. It is practical and safe to use around the lateral recess and the foramen. The use of the thin ultrasonic bone shaver tip (1.8 mm) makes it safe to insert it into very narrow epidural space. In severe lumbar stenosis cases, we performed a total laminectomy with extended foraminotomy. The first step in laminectomy is to create a safe epidural space. It is an advantage to use UBShaver in very narrow epidural spaces where the Kerrison rongeur is not applicable. Once a window is opened, laminectomy is carried out with the Kerrison rongeurs or high-speed drills. After performing the laminectomy, the ligamentum flavum is exposed and the foraminotomy is started with removing the medial surface of the inferior articular process with the UBShaver. At this point it is crucial not to excise the ligamentum flavum too early, since it protects the dura underneath. Cotton patties are not needed as long as the ligamentum flavum is intact. Then the medial aspect of the superior articular process is exposed and removed with the UBShaver. The decompression of the neural foramen is continued by removing the lateral aspect of the superior articular process. If necessary, the inferolateral wall of the pedicle can also be thinned. The last step to decompress the foraminal exit zone is when the pars interarticularis is removed with the UBShaver.

**Figure 2:**
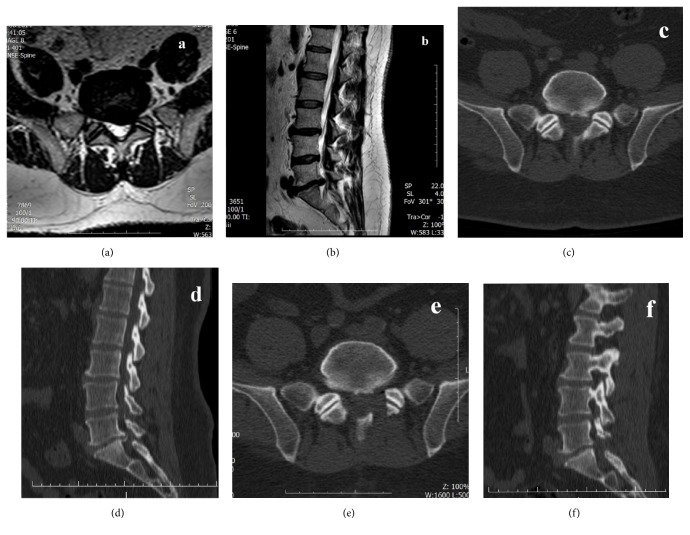
Preoperative (a) axial and (b) sagittal MRI with T2 weighted image and (c) axial and (d) sagittal CT images of a calcified left L5-S1 level disc herniation causing lateral recess syndrome and postoperative (e) axial and (f) sagittal CT images demonstrating decompressed foramen with limited foraminotomy.

In lateral lumbar stenosis surgery, we performed a hemilaminectomy with limited foraminotomy (Figure 2). Hemilaminectomy is performed in a similar fashion as described above. In limited foraminotomy, the medial surface of the inferior articular process and the superior articular process is removed, and the ligamentum flavum is excised. To be able to widen the lumbar foramen the decompression is continued with the medial part of the pars interarticularis. The anterior cortex of the pars interarticularis is removed along the nerve root, decompressing the foraminal exit zone, in cases with very lateral stenosis. In limited foraminotomy, the posterior surface of the pars interarticularis is kept intact with at least a five-millimeter thickness for stability reasons.

In far lateral disc herniation, the lateral foraminotomy was performed with a posterolateral muscle splitting approach [11]. The pedicle-transverse process junction and the intertransverse ligament are exposed. The lateral border of pars interarticularis together with the superolateral aspect of the superior facet of the caudal vertebra is excised by using the UBShaver to decompress the foramen laterally.

In recurrent disc herniation cases, the key step is to find the bony edge of the previous hemilaminectomy or foraminotomy site. The UBShaver is introduced to widen the foramen and to find the “untouched” dura through the fibrotic tissue.

#### 2.3.2. Thoracic Posterior Applications

In cases of intradural or intramedullary spinal tumors the UBShaver can be used safely for the laminectomy at the thoracic level without putting any download pressure to the spinal cord. In vertebral body fractures at this level, in order to expose the pedicle, the UBShaver can also be used efficiently in similar fashion that is previously described in the lumbar section.

#### 2.3.3. Cervical Posterior Applications

In the posterior cervical approach for an extensive ossified posterior longitudinal ligament and/or a severe canal stenosis, we have performed an open door laminoplasty. The UBShaver is useful when removing the inner cortex of the lamina without damaging the epidural venous plexus and the nerve roots.

In foramen magnum decompression for a Chiari I malformation the decompression of the lateral edge of the foramen magnum is often not easy to perform, when using rongeurs and drills, due to the dull angle of the edge and fear of injuring the vertebral artery. With the UBShaver, the lateral edge of the foramen can easily be removed for foramen magnum decompression without any risk of damaging the vertebral arteries or the epidural venous plexus.

#### 2.3.4. Cervical Anterior Applications

**Figure 3:**
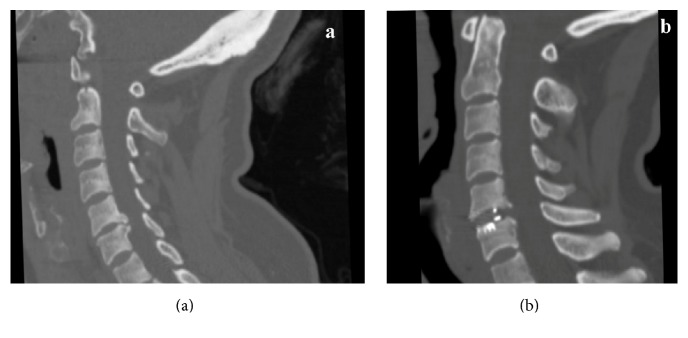
(a) Preoperative and (b) postoperative sagittal CT images of a C5-6 disc herniation with osteophytes.

In an anterior approach for cervical disc herniation, osteophytes in the posterior surface of the end plates make the operation technically demanding. Removal of the osteophytes on both sides, close to the foramen, was done successfully with the UBShaver (Figure 3). However, it was challenging to remove the midline osteophytes because the hand piece has a short extension (57 cm) and blocks the microscopic vision. This problem was overcome by expanding the disc level with a Caspar distractor. However, we recommend the use of a longer tip or a tailor tip shaver for osteophytectomies in the anterior cervical approach.

In cases of cervical tumors or fractures, in which the corpectomy is needed, the UBShaver is the main tool to complete the vertebral body bone removal adjacent to the posterior longitudinal ligament.

## 3. Results

In the 307 cases we found a predominance of women (182 (59%)). The mean values of the operation time differ from 73 minutes to 196 minutes for different lumbar pathologies, 169 minutes to 213 minutes for thoracic pathologies, and 107 minutes to 154 minutes for cervical pathologies.

The need for blood transfusion was low. Most blood transfusions (500 mL) were needed for lumbar stenosis cases (136/307, 44%). For thoracic (12/307, 3%) and for cervical (3/307, 1%) vertebral fractures the need for blood transfusion was low.

The mean preoperative ODI scores in lumbar and thoracal cases were highest in thoracal vertebrae fracture cases (48%). At the follow-up, one year after surgery, ODI score dropped to 18% in lumbar stenosis cases and to 8% in lumbar disc herniation cases. The preoperative NDI score in cervical cases was the highest at 68% in cervical vertebrae trauma cases and it dropped to 34% at follow-up (Table 1).

There were five cases of dural tear among the lumbar cases. Four of the patients with dural tear were severe stenotic cases. They had calcified dura mater at the midline laminectomy stage and no cerebrospinal fluid support to emulsify the oscillation power from the ultrasound device. The remaining one patient with a dural tear had a lateral recess syndrome. It occurred at the foraminotomy stage in which the ligamentum flavum was excised before the bony removal was done. All cases occurred in the first 50 cases when we started to use this instrument as an assisting tool.

There was no postoperative infection or wound healing problems, and we had no reoperations related to these morbidities. There were also no neurological deficits related to the application of this tool.

## 4. Discussion

We have experienced that the ultrasonic bone curette is a useful instrument in very narrow epidural spaces, while avoiding excessive heat production, minimizing blood loss and operating time, and therefore limiting the risk of mechanical injury. We can recommend the device for various spinal surgery fields and especially as the only tool in limited foraminotomies.

We have used the microhook shaver tip (1.8 mm) which can be easily introduced to very narrow epidural spaces. The shaver tip osteotome causes fragmentation and cavitation in the bone [17]. The hand piece is light weighted, can be readily manipulated with one hand, and is cooled by automatic irrigation of physiologic saline when applied [13].

There are several publications presenting successful applications of ultrasonic bone curette with various hand pieces (Table 2). It has been used for several indications from tumors to vascular malformations and for several levels from foramen magnum to sacral level [12, 14–19]. It is presented as either a primary tool for minimally invasive surgery or an assisting tool for decompression and stabilization. However, there are some concerns related to its application regarding heat generation and prolonged operating time. The heat generated by an ultrasonic device on bone has been reported to be no more than that generated by high-speed drills [19]. It has also been shown that there is no danger of causing thermal injury to the surrounding important neural and vascular structures [12]. However, one study reported a spinal cord injury caused by prolonged application of the instrument at one location [5]. Some authors have claimed that somewhat longer operation time was needed during application of the ultrasonic bone curettes when high amount of bone bulk was to be removed [8, 9]. They presented a mean operation time of 243 minutes for hemilaminectomy in mostly spinal tumor cases [8]. In our series, 118 (38%) patients with lumbar disc pathologies had a mean operating time of 75 minutes with the device as the primary tool. In 158 (51%) cases with cervical and lumbar stenosis the UBShaver was used as an assisting tool and the surgery was completed in approximately 180 minutes. Our figures were lower compared to data presented in literature and the surgeons felt that it reduced the time spent in the operating theater.

The incidence of injury to the dura mater has been reported to be similar or even lower by the use of the ultrasonic bone curette when compared with air drill systems [3, 4, 18]. Many have reported no complications with dural tear when using ultrasonic bone curettes [5, 6, 8, 9], and others have reported an incidence of dural tears between 1.6% and 9.8% [16, 19, 20]. In our series, with high number of cases, we had only five cases with dural tears (1.6%). Four of our patients with dural tear were severe stenotic cases with calcified dura mater which had no cerebrospinal fluid support to emulsify the oscillation power from the ultrasound device. The remaining one patient had a lateral recess syndrome. However, all these five patients were among the first 50 cases in our series and each surgeon (authors: Derya Burcu Hazer, Barış Yaşar, and Aytaç Akbaş) has experienced at least one dural tear within their first 15–20 cases. In these cases of lumbar stenosis the instrument was used as an assisting tool. Ligamentum flavum was removed in the beginning of the surgery and the dura was left bare. Additionally, in these first cases we have applied the instrument in a more “vertical plane.” After these 50 cases, we have spared the ligamentum flavum to limit the risk of dural tear. We have also applied the instrument in a more horizontal plane to decrease the thickness of the bone rather than cutting it (Supplementary Video, in Supplementary Material available online at http://dx.doi.org/10.1155/2016/8428530). We can claim that surgeons who want to start using the instrument would need about 15–20 cases to reach their own learning curve and feel comfortable with the device as an assisting tool. We recommend that due to the depth of the surgical field and a more narrow working space the application of the instrument in anterior cervical cases should be performed after achieving the learning curve.

Some safety aspects have been pointed out by others. The energy of the instrument should be decreased to 60% and the instrument should not stay on one point more than 10 seconds [6, 21]. In laminectomy cases, the lamina can be thinned until a thin bone lamella is remaining and then cut with the bone curette [6].

To our knowledge the main reason for an iatrogenic dural tear when using the UBShaver is the application of the tip directly to one point for a long time. Additionally, with our described limited foraminotomy technique the posterior supporting elements, such as the supraspinous and the interspinous ligaments and the spinous process, can be preserved [11]. We recommend intermittent usage of the device, cotton protection with constant irrigation, and use of an operating microscope when necessary.

In studies comparing the application of ultrasonic bone remover and high-speed drill in spinal cases, the hospital stay, the duration of the surgery, and the blood loss were found to be significantly lower with the ultrasonic bone remover [3, 18]. In our series we have also detected low blood loss and most of our cases were completed in 90 minutes. We have also found that the UBShaver has a local haemostatic effect and reduces the bone bleeding, which presented as no blood transfusion used in one-level lumbar surgery.

The clinical follow-up with the ODI and the NDI scores showed a decrease in the ODI score one year after surgery in lumbar stenosis cases (42% to 18%), and a similar major decrease was detected in the NDI score in cervical stenosis cases (58% to 16%). Our scores are similar to those found recently in the literature when using the conventional surgical method [20, 22]. No others, using the technique with ultrasonic bone removers, have been presenting data with a clinical improvement. A limitation of this study is the lack of a comparison group operated on with conventional techniques. However, we still believe that the equipment can improve spine surgery by decreasing the operating time and lowering the complication rates.

All new surgical devices have a learning curve. We would like to highlight some technical key points when using the UBShaver in spine surgery that might help surgeons who wish to use the equipment:

1. Intermittent usage and cotton protection with constant irrigation are mandatory.
2. In lumbar surgery the ligamentum flavum can be left until the end of the bone removal process, since it acts as a natural barrier.
3. The ultrasonic bone remover with a shaver tip is very effective in foraminotomy when a lateral recess syndrome or far lateral stenosis is present. It can be used as the only instrument when performing a limited foraminotomy, without causing any instability.
4. In anterior approaches to the cervical spine, especially when removing midline posterior osteophytes, a longer attachment and a tailor shaver tip should be used.
5. In thoracic vertebral fractures the device can be used to expose the pedicle in dislocated vertebral fractures and performing laminectomies without applying any download pressure on the spinal cord.

## 5. Conclusion

The ultrasonic bone curette is a useful instrument, limiting heat production and decreasing the risk of mechanical injury, when applied close to dura mater or other neural tissues. We recommend the device for application alone in limited foraminotomy and in various other spinal surgery fields as a complement to high-speed drills and other instruments. However, further refinement of this tool is necessary before it can be used in anterior osteophytectomy in the cervical spine surgery. We suggest that the tips should be longer and have an ability to work at wider angles. The hand pieces should be made slender for easier maneuverability.

## Supplementary Material

A video demonstration of decompression of foramen and removal of pars interarticularis at the right L4-5 level with ultrasonic bone shaver is presented.

## Figures and Tables

**Table 1:** The number of patients, gender, age, surgical data, and follow-up data are given for each pathology type operated with the aid of Misonix ultrasonic bone shaver. Values are given as mean (range) when applicable.

Pathology level	Type of pathology	Number of cases (*n*)	Gender M/F (*n*)	Age at surgery (years)	Surgical technique	Operation duration (min)	Blood transfusion (mL)	Dural tear (*n*)	Preop ODI (%)	Postop ODI (%)	Preop NDI (%)	Postop NDI (%)
Lumbar	Unstable lumbar central and/or lateral stenosis (>1 level)	136	54/82	68 (45–71)	Laminectomy + extended foraminotomy + posterior stabilization + fusion	196 (90–257)	500 (250–750)	4	42 (32–56)	18% (6–28)	IA	IA
	Lateral recess syndrome	70	23/47	52 (31–68)	Hemilaminectomy + limited foraminotomy	75 (55–120)	0	1	38 (24–45)	16% (6–24)	IA	IA
	Far lateral disc	16	7/9	63 (43–72)	Lateral foraminotomy	73 (42–135)	0	0	25 (12–45)	8 (4–12)	IA	IA
	Recurrent disc herniation	32	14/18	58 (30–69)	Revision hemilaminectomy + limited foraminotomy	75 (64–134)	0	0	28 (14–35)	8 (2–12)	IA	IA

Thoracic	Spinal intramedullary tumors	5	1/4	46 (25–65)	Laminectomy	213 (75–278)	0 (0–250)	0	46 (32–56)	25 (8–36)	IA	IA
	Traumatic vertebral body fractures	12	8/4	57 (28–76)	Laminectomy and exposing pedicle anatomy	214 (97–256)	500 (0–750)	0	49 (42–56)	38 (12–48)	IA	IA

Cervical	Cervical stenosis	22	12/10	71 (65–78)	Posterior laminoplasty	154 (100–195)	500 (0–750)	0	IA	IA	58 (25–75)	16 (8–26)
	Chiari malformation	3	1/2	38 (29–45)	C1 laminectomy + foramen magnum decompression	143 (98–184)	250 (0–500)	0	IA	IA	12 (4–25)	8 (4–24)
	Cervical vertebral body fracture	3	2/1	47 (43–68)	Corpectomy + anterior fusion	137 (116–174)	500 (250–750)	0	IA	IA	68 (23–80)	34 (8–45)
	Cervical disc, calcified, and osteophytes	8	3/5	65 (47–73)	Resection of calcified disc and end plates	107 (65–146)	0	0	IA	IA	15 (2–18)	9 (3–19)

^*∗*^Preop ODI: preoperative Oswestry Disability Index; postop ODI: postoperative Oswestry Disability Index; preop NDI: preoperative Neck Disability Index; postop NDI: postoperative Neck Disability Index; hr: hour; min: minutes; IA: inapplicable.

**Table 2:** The literature review of the application of the ultrasonic bone remover.

	Instrument	Number of cases	Level of the pathology	Technique	Complication
Nakagawa et al., 2005 [4]	UBShaver	76	Chiari malformation, cervical, thoracic, lumbar	Microsurgical decompression	None

Schaller et al., 2005 [12]	UBC-scalpel	2	Tethered cord, lumbar	Laminectomy	None

Kim et al., 2006 [5]	UBShaver	546	Chiari malformation, cervical, thoracic, lumbar	Foraminotomy, lateral recess exposure	5 dural tears, 1 transient spinal cord injury

Nakase et al., 2006 [7]	UBShaver	98	Chiari malformation, cervical, thoracic, lumbar	Microsurgical decompression	None

Ito et al., 2009 [13]	UBC-scalpel	12	Lumbar	Laminoplastic laminotomy and hemilaminotomy	1 dural tear

Landi et al., 2011 [14]	UBC-not specified	1	Thoracal, calcified disc	Transversoarthropediculectomy	None

Matsuoka et al., 2012 [8]	UBC-scalpel	33	Chiari malformation, cervical, thoracic, lumbar	Recapping hemilaminoplasty	None

Morimoto et al., 2012 [10]	UBC-scalpel	26	Lumbar	Medial fenestration	None

Kim et al., 2012 [15]	UBC-unknown	Not detailed	Cervical	Foraminal decompression in cervical anterior fusion	Not detailed

Bydon et al., 2013 [3]	UBC-scalpel	88	Thoracolumbar	Microsurgical decompression	5 dural tears

Hu et al., 2013 [16]	UBC-scalpel	128	Chiari malformation, cervical, thoracic, lumbar	Facetectomy, laminotomy, laminectomy, en bloc resection, the Smith-Petersen osteotomy, pedicle subtraction osteotomy	2 dural tears

Al-Mahfoudh et al., 2014 [17]	UBC-scalpel	62	Chiari malformation, cervical, thoracic, lumbar	Laminotomy, corpectomies	1 dural tear

Bydon et al., 2014 [2]	UBC-scalpel	10	Thoracolumbar	Microsurgical decompression	3 dural tears

Onen et al., 2015 [18]	UBC-scalpel	23	Cervical myelopathy	Cervical laminectomy	1 C5 radiculopathy

Hazer et al., 2016 (this study)	UBShaver	307	Chiari malformation, cervical, thoracic, lumbar	Facetectomy, laminotomy, laminectomy, en bloc resection, pedicle subtraction osteotomy	5 dural tears

UBC: ultrasonic bone curette.
